# Cross shard leader accountability protocol based on two phase atomic commit

**DOI:** 10.1038/s41598-024-64945-1

**Published:** 2024-06-28

**Authors:** Zhiqiang Du, Wendong Zhang, Liangxin Liu, Yanfang Fu

**Affiliations:** Xi’an Technological University, Xian, 710021 China

**Keywords:** Computer science, Information technology

## Abstract

Sharding blockchain is a technology designed to improve the performance and scalability of traditional blockchain systems. However, due to its design, communication between shards depends on shard leaders for transmitting information, while shard members are unable to detect communication activities between shards. Consequently, Byzantine nodes can act as shard leaders, engaging in malicious behaviors to disrupt message transmission. To address these issues, we propose the Cross shard leader accountability protocol (CSLAP), which is based on the two-phase atomic commit protocol (2PC). CSLAP employs byzantine broadcast/byzantine agreement (BB/BA) for Byzantine fault tolerance to generate cross-shard leader re-election certificates, thereby reducing the impact of shard leaders on inter-shard communication. It also uses Round-robin mechanism to facilitate leader re-election. Moreover, we demonstrate that CSLAP maintains the security and liveness of sharding transactions while providing lower communication latency. Finally, we conducted an experimental comparison between CSLAP and other cross-shard protocols. The results indicate that CSLAP exhibits superior performance in reducing communication latency.

## Introduction

A blockchain^1^ is essentially a decentralized database. It is a new application mode of distributed data storage^2,3^, point-to-point transmission, consensus mechanisms, encryption algorithms and other computer technologies^4,5^. However, for blockchains to be more widely used, the transaction throughput must be improved, and the confirmation delay should be decreased. Sharding is one of the key methods for solving the blockchain scalability problem, and an increasing number of systems are implementing sharding blockchains^6,7^. The key idea behind this approach is to create groups of nodes (or shards) that process only a subset of all transactions and system states, relying on the classic Byzantine fault tolerance protocols (BFTs) to achieve intra-shard consensus. The main purpose of intra-shard consensus is to process transactions within a shard. Therefore, the design of a consensus algorithm within a shard plays a vital role in the efficiency of transaction processing. The consensus algorithm needs to be able to efficiently process transactions within a shard and coordinate with other shards to complete cross-shard transactions, and this process requires the consensus algorithm in the shard to have a high degree of reliability and security to ensure stable network operations. Additionally, some sharding blockchains use reference committees^8^, so intra-shard consensus is also used to confirm the committee membership list. These sharding blockchain systems achieve optimal performance and scalability because: (i) Nonconflicting transactions can be processed in parallel by multiple shards; and (ii) The system can scale by adding new shards. This separation of transaction processing across shards is not completely “clean”; a transaction may depend on data managed by multiple shards, requiring the extra step of cross-shard consensus among related shards.

Atomic commit protocols (such as the two-phase atomic commit protocol (2PC)) typically operate on all relevant shards to ensure that cross-shard transactions are accepted by all or none of the shards.

In the 2PC protocol^9^, communication between shards is crucial. The 2PC protocol consists of a preparation phase and a commit phase^9^. During the preparation phase, the shard runs an in-shard consensus algorithm to prove whether the transaction input is available. We call this the availability certificate (*AC*). To reduce the complexity of communication, shards often elect a leader to act as a representative for communicating with other shards. However, centralizing trust in one node can lead to malicious behaviour. We introduce cross-transaction censorship attacks in the sharding blockchain, where an attacker can attack the leader of a related shard in a transaction, such as the BFT initiator of each shard in a Chainspace^10^; After the shard leader is attacked and becomes a malicious leader, that malicious leader normally participates in and executes the BFT consensus algorithm within the shard (such as the PBFT, HotStuff, or Marlin); however, when cross-shard transactions are involved, the *AC* needs to be generated and sent to the relevant shards, and the malicious leader will not send *AC* to other shards related to the transaction. If the other shards do not receive all *AC*, they cannot agree on every part of the transaction, and the transaction will be permanently delayed. Although the intra-BFT consensus within a shard does not affect its liveness and security, the loss of agreement activity between shards still compromises the overall activity of the sharding blockchain. An attack can be executed even if the Byzantine security assumption^11^ is met, and this attack wastes system resources.

There are currently two critical cross-shard consensus methods: (i) client-led cross-shard consensus and (ii) shared-led cross-shard consensus^12^. An attack on this type of consensus suits the shard-led cross-shard consensus and does not require coordination from external entities. Based on our analysis of performance trade-offs and vulnerabilities related to cross-transaction censorship in current shard-led cross-shard protocols, we propose the CSLAP protocol. The CSLAP we propose is general and applicable to sharding blockchain systems that use shard-led cross-shard consensus, such as RapidChain^13^, which utilizes the process of a 2PC to achieve high performance and scalability. RapidChain also combines the 2PC process with other methods to resist cross-transaction censorship attacks to achieve a more efficient protocol. The CSLAP identifies malicious shard leader behaviour when it does not receive the information that would allow it to perform the conversion of malicious leaders between shards and shards in the 2PC^9^. In the case of malicious behaviour, replacing the malicious leader node is the quickest solution compared to other remedial actions.

We implemented the CSLAP prototype in the Go language and compared it with other cross-transaction censorship attack defence protocols. The results show that the delay of our protocol is lower.

This study makes the following main contributions: (i) Cross-transaction censorship attacks against a shard-led cross-shard consensus protocol were studied. Then, we introduced the classic sharding blockchain protocol and the latest cross-transaction review attack defence protocol. (ii) A new CSLAP that integrates our proposed defences to achieve resilience against cross-transaction censorship attacks without compromising the 2PC protocol was designed. (iii) The Byzantine prototype was implemented, and its performance was evaluated on real distributed node sets. Then, it was compared with previous protocols such as the CSVC^14^ and the CSVC in a flexible sharing blockchain protocol (FS_CSVC)^15^. The results showed that the latency of the CSLAP protocol was the lowest.

## Related work

Among cross-shard blockchains, ELASTICO^16^ was the first public, decentralized, shard-based blockchain system. In ELASTICO, each shard is responsible for verifying a set of transactions and reaching a consensus based on the practical Byzantine fault tolerance (PBFT) algorithm. Afterwards, the final shard verifies all of the transactions received from the shards and includes them in a global block, which is then broadcast to all of the nodes in the system and stored. In RapidChain^13^, clients no longer need to request asset proofs from each input committee. Instead, they only need to send the transaction to any committee, and then, the transaction is routed to the output committee through the inter-committee routing protocol. In the OmniLedger solution^17^, the client initiates a request to the input committee, expecting each input committee to provide proof of acceptance for its respective assets. If the client does not receive all of the asset proofs and does not unlock the command, it assumes that the shards are honest and will not fail. Eventually, all of the messages are received, and a BFT consensus is reached. OptChain^18^ includes an optimal transaction placement strategy by learning past transaction patterns, while BrokerChain^19^ divides the account state graph through Metis, reducing cross-shard transactions based on the account model. Both models aim to reduce the number of transactions across shards and maintain load balancing across shards by appropriately distributing transactions to shards. Chainspace^10^ employs a shard-led cross-shard consensus protocol called SBAC, wherein the client submits a transaction to the input shard. Each shard internally executes a BFT protocol, which enables it to make a temporary determination regarding the local acceptance or termination of the transaction. Subsequently, the shard broadcasts its local decision (preaccept(T) or preabort(T)) to other shards through the BFT initiator.

The above protocol assumes that the final result of each shard is honest and that the shard leader is honestly sending the generated information to the relevant nodes during the cross-shard process. In general, a malicious node in a shard can change the leader by trying to switch protocols, but members in a shard are usually unaware of the information between shards. Pyramid^20^ is a blockchain based on hierarchical sharding in which some nodes can store multiple shards and process cross-shard transactions involving those shards. Since nodes may be waiting for resources to be released during the block commit phase, system deadlocks can easily occur. PolyShard^21^ applies coded computation to sharding for linear scalability and security. It splits data into coded shards, enabling parallel processing and recovery of damaged shards. Ren et al.^22^ proposed root graph placement to identify the shards that are most suitable for transactions based on interactions between the most recent transactions and historical transaction s and designed two techniques to mitigate the impact of the remaining cross-shard transactions on the system performance. One technique parallelizes dependent transaction validation with atomic commit protocols, and the other incorporates atomic commit protocols. X-shard^23^ is a cross-shard transaction scheme for blockchain systems that uses an optimistic strategy to handle low-latency multiple input multiple output (MIMO) cross-shard transactions. Transactions are assumed to be valid and only verified when they are about to be committed to the output shard. Cross-shard transactions are processed through a gateway account managed by the board. Each cross-shard transaction is broken down into multiple intra-shard sub-transactions that transfer coins to the gateway account in the input shard. Input shards process sub-transactions in parallel without waiting for other shards. If cross-shard transaction verification fails, the transaction is rolled back to ensure atomicity. The above protocols can reduce the proportion of cross-shard transactions or improve the delay they cause through parallelization , transaction merging and other technical means. As^24^ noted, most of the previous studies were based on smaller variants of 2PC for cross-shard transactions.

In the description of cross-shard consensus attacks for Byzcuit^25^, which was designed by Alberto et al., the attackers resist the replay attacks of both the shard-led and client-led cross-shard consensus protocols. For cross-transaction review attacks, after the output leader does not receive a relevant shard certificate, the secure cross-shard view-change protocol^14^ (CSVC) proposed by Liu et al attempts to perform intra-shard consensus to generate a cross-shard view-change certificate to replace the view. However, the protocol’s design cannot accurately detect malicious behaviour exhibited by the leader within its own shard or within an input shard. The process of re-inquiry by shard members can only ensure the normal execution of the protocol, which increases the burden in terms of delay, and malicious leadership styles cannot be accurately identified. Liu referenced and improved the cross-shard view-change protocol in a flexible sharding blockchain protocol^15^ in 2023 (FS_CSVC). The two cross-preparation and cross-commit construction stages are used to generate cross-shard view-change certificates. This protocol incorporates the members processes by repeatedly asking for certificate information. However, the process of re-inquiry still exists, and the problems mentioned above still exist.

Therefore, this paper proposes the CSLAP, which reduces the query process and ensures that leaders’ malicious behaviour is detected at every step, thus reducing latency and improving security across shared consensus.

## System model and overview

### System model

#### Transaction (*tx*)

*tx* refers to an operation or event recorded and verified on the blockchain network. Transactions typically involve the transfer of data, assets, or value, which serve as the fundamental building blocks of a blockchain system. A transaction consists of multiple inputs and outputs:

Inputs: These define the source of *tx*, which could be outputs from previous *tx* or other types of digital assets.

Outputs: These represent the destination of *tx*.

#### Honest shard

 We assume that all of the shards in the protocol are honest. An honest shard means that the proportion of honest members within it meets the target security threshold determined by the shard’s internal consensus algorithm. Additionally, the shard reorganization process does not impact the assumption of shard honesty.

In a sharding environment, the input and output of a transaction can be in different shards. We refer to the shard that manages the input as the input shard ($shard_{in_1}$,..., $shard_{in_n}$) and the shard that manages the output as the output shard ($shard_{out_1}$,..., $shard_{out_n}$). Sometimes, if a transaction’s partial input and output are in the same shard, that shard can simultaneously be both an input and an output shard.

We indicate that $T\{(I_1,..., I_x) \rightarrow (O_1,...,O_y),I_x\in shard_ {in_x},O_y\in shard_ {out_y} \}$. For example, $T\{(I_1, I_2)\rightarrow (O_1, O_2, O_3)\}$ indicates a transaction with two inputs $I_1,I_2$ and three outputs $O_1, O_2, O_3$, including the input divided $shard_ {in_1}, shard_ {in_2}$ and the output divided $shard_ {out_1}, shard_ {out_2}, shard_ {out_3}$, $shard_ {in_1}$ and $shard_ {out_1}$, which are the same shard; however, it is easier to describe the different shard identities during the protocol operation.

#### Node

 Let $R_{in}$={$r_{in_1}$,...,$r_{in_i}$,...,$r_{in_n}\}$ and $R_{out}$ = {$r_{out_1}$,$r_{out_2}$...,$r_{out_i}$,...,$r_{out_n}\}$ be the set of *n* nodes in $shard_{in}$ and $shard_{out}$. Each node $r_{out_i}$ or $r_{in_i}$ has a key pair ($sk_i$, $pk_i$) and is uniquely identified by its public key $pk_i$ in the system. We assume a public key infrastructure (PKI) that knows the public keys of all nodes to prevent identity spoofing.

In this system, nodes are classified based on their behaviour towards the protocol as follows:

Honest nodes: These nodes adhere to the protocol, following the predefined rules and processes with integrity. Honest nodes ensure the system’s reliability by conducting operations transparently and without deviation.

Malicious nodes: These nodes deviate from the protocol, engaging in behaviours that contradict or undermine the established rules. Malicious nodes may attempt to manipulate the system for personal gain or to disrupt the consensus process. They represent a risk to the system’s security and can cause inconsistency or data corruption.

#### Adversary model

 An adversary can destroy *f* of *n* nodes per-shard, where $n\ge 2f+1$ and the remaining nodes are correct. Therefore, the number of honest nodes is greater than *n*/2 to ensure an honest majority within each shard. The adversary is static, i.e. it chooses a set of *f* nodes to destroy at the beginning of the protocol and cannot choose to destroy other nodes afterwards.

#### Quorum certificate

 There is a group of voting signatures that form the node’s quorum certificate (QC), and the QC is composed of the voting rights of nodes 1/2 or more in the shard. A QC serves as a crucial component in blockchain governance and consensus mechanisms, ensuring that an action or block has received sufficient approval from the participating nodes. It acts as a proof of majority support, validating the authenticity and consensus behind a decision or block addition.

#### Network model

 We adopt a synchronous network model in which any message sent by an honest node at time *t* arrives at another node before time $t+\Delta$, where $\Delta$ is the known network maximum delay. The opponent can pass the message delay to a known upper bound $\Delta$.

### Security properties

#### Definition 1

(Consistency) In sharding blockchains, honest members reach a consensus on a transaction and will not commit to two conflicting transactions.

#### Definition 2

(Liveness) For any valid *tx* submitted by a client, after a certain period, all relevant shards finally decide whether to execute accept(T) or abort(T).

#### Definition 3

(Cross-shard transaction censorship attack) Let $tx=T\{(I_1,...,I_x) \rightarrow (O_1,...,O_y)$ be a cross-shard transaction where $I^j(j\in [1,x])\in shard_{in_j}$. Let $L_{j}$ behave as the leader of $shard_{in_j}$. If $L_{in_j}$ pretends to be honest in $shard_{in_j}$ but does not transfer or send invalid *AC* between related shards, then $L_{in_j}$ is judged to launch a cross-shard transaction censorship attack.

**Figure 1:**
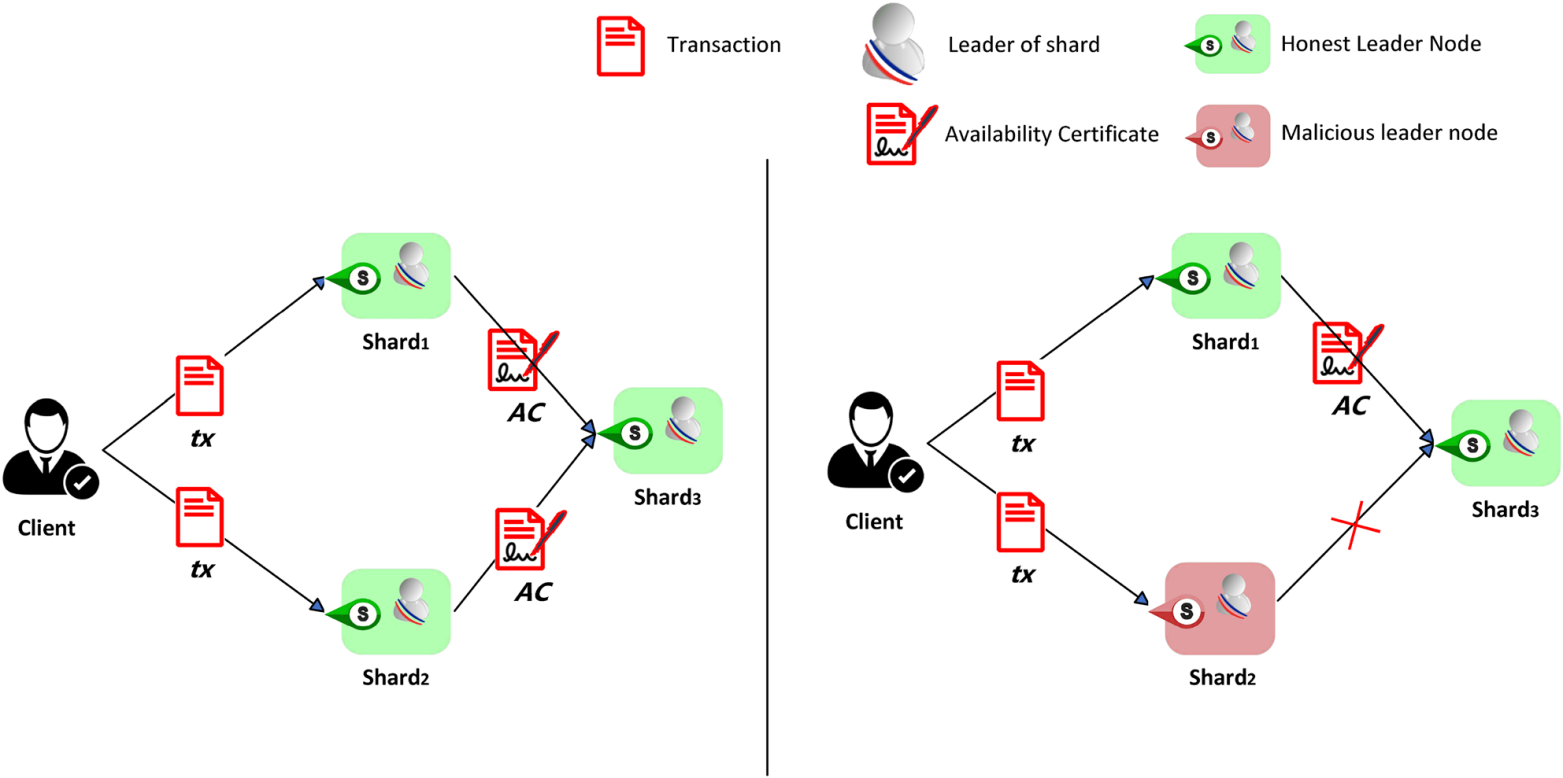
Cross-transaction censorship attack display.

Figure 1 briefly shows an example of an attack across attempts at censorship, depicting various situations in the 2PC phase for a *tx* with two inputs and three outputs.

**Table 1:** Highlighted rows in the table represent correct executions of 2PC (i.e. without an attacker); other rows represent incorrect executions due to cross-transaction censorship attacks.

	Prepare phase of 2PC		Commit phase of 2PC		
	$Shard_{in_1}$ (potential victim)	$Shard_{in_2}$ (potential victim)	$Shard_{out_1}$ (potential victim)	$Shard_{out_2}$ (potential victim)	$Shard_{out_3}$ (potential victim)
1	pre-accept(T) lock x1	pre-accept(T) lock x2	accept(T) inactivate x1	accept(T) inactivate x2	accept(T) -
2	pre-accept(T) lock x1	pre-accept(T) lock x2;withhold	abort(T) unlock x1	abort(T) unlock x2	abort(T) -
3	pre-accept(T) lock x1;withhold	pre-accept(T)ock x2	abort(T) unlock x1	abort(T) unlock x2	abort(T) -
4	pre-abort(T) -	pre-abort(T) -	abort(T) -	abort(T) -	abort(T) -
5	pre-abort(T) -	pre-accept(T) lock x2	abort(T) -	abort(T) unlock x2	abort(T) -
6	pre-abort(T) -	pre-accept(T) lock x2;withhold	abort(T) -	abort(T) unlock x2	abort(T) -
7	pre-abort(T) withhold	pre-accept(T) lock x2	abort(T) -	abort(T) unlock x2	abort(T) -
8	pre-accept(T) lock x1	pre-abort(T) -	abort(T) unlock x1	abort(T) -	abort(T) -
9	pre-accept(T) lock x1	pre-abort(T) withhold	abort(T) unlock x1	abort(T) -	abort(T) -
10	pre-accept(T) lock x1;withhold	pre-abort(T) -	abort(T) unlock x1	abort(T) -	abort(T) -

The content of each row can be divided into two sub-lines; the first sub-line shows the protocol messages sent by the shard, and the second sub-line represents the local shard operations after sending these messages. The first two columns represent the state of the input shard against the transaction at the end of the first phase of 2PC. The last three columns represent the final state of the output shard for the transaction after the second phase of 2PC ends. For example, (line 2) $Shard_{in_2}$ is meant to provide pre-accept(T); however, due to the leader’s withholding behaviour, pre-accept(T) was not received by any input fragments during the second stage. This issue leads to honest deals being rejected, and if the transaction execution fails, all shard operations are rolled back.

The two inputs also serve as outputs for receiving acceptance or rejection information from the set coordinator. Here, the output $shard_{out_3}$ acts as the coordinator. After receiving *AC* from the input shards $shard_{in_1}$ and $shard_{in_2}$, the blockchain system performs verification and sends acceptance or rejection information back to $shard_{in_1}$ and $shard_{in_2}$. Table 1 shows the cross-censorship transaction attack behaviour in the 2PC phase. Preaccept(T) means that an input shard temporarily writes changes locally; preabort(T) indicates that the write failed. Lock refers to locking a specific related object of the transaction; accept(T) means that the coordinator has received confirmation of the transaction, while abort(T) means that the coordinator has sent information indicating that the transaction has been rejected. Inactivated refers to permanently confirming changes and releasing related resource information, and unlocked refers to unlocking resources and rolling back the local writes.

### Performance metrics

The CSLAP protocol involves a performance metric (the delay between finding a maliciously behaving leader and switching to an honest leader):

- Latency (ms): latency for submitting cross-shard transactions.
- Throughput (bytes/s): the number of bytes processed per second.

## CSLAP:Cross shard leader accountability protocol

### Protocol overview

The CSLAP allows the coordinator shard $Shard_{out}$ to observe the malicious behaviour of $Shard_{in}$’s leader. Subsequently, the CSLAP generates a shard $Shard_{in}$ through the consensus algorithm proof related to the leader’s malicious behaviour and sends it to the members of $Shard_{in}$. After the members of $Shard_{in}$ judge the authenticity of the cross-shard leader re-election quorum certificate (CSLAP-QC) sent by $Shard_{out}$, the members of $Shard_{in}$ use the round-robin algorithm to replace the malicious leader. Here, we use the BFT protocol based on the two primitives, BB/BA. Using a BFT protocol based on the BB requires protection against malicious behaviour by shard leaders who send cross-shard certificates. The BFT protocol based on the BA can omit the leader’s negative behaviour.

### Primitives

#### **Round-robin leader election**^26^

The round-robin approach allows nodes to elect a leader for each epoch. The round-robin algorithm takes the last block as input and outputs a random leader from all nodes excluding the last *f* and misbehaving leaders.

#### Byzantine broadcast

 A Byzantine broadcast is a protocol that allows a certain node (called the sender) to consistently distribute a message among *n* intra-shard nodes, in which up to *f* nodes are Byzantine.

##### Definition 4

(Byzantine Broadcast^27^) A Byzantine broadcast satisfies the following properties:

- **(Agreement)** If two honest nodes commit values *B* and $B'$, respectively, then *B* =$B'$.
- **(Termination)** All honest replicas eventually commit and terminate.
- **(Validity)** If the designated sender is honest, then all honest nodes commit on the sender’s value.

#### Byzantine agreement

The Byzantine agreement is a protocol that allows certain nonfaulty (honest) replicas to decide on the same intra-shard output, in which up to *f* nodes are Byzantine.

##### Definition 5

(Byzantine Agreement^27^) The Byzantine agreement satisfies the following properties:

- **(Agreement)** If two honest nodes commit values *B* and $B'$, respectively, then *B* =$B'$.
- **(Termination)** All honest replicas eventually commit and terminate.
- **(Validity)** If all honest replicas have the same input value, then all honest replicas commit on the value.

##### Definition 6

(Byzantine Fault Toleranc^28^) The BFT is a pivotal algorithm in distributed computing systems that is designed to achieve consensus in networks in which some nodes may fail or act maliciously. The BFT addresses the challenge of ensuring that a distributed system continues to operate reliably and reaches consistent decisions even when a subset of nodes experiences faults or exhibits hostile behaviour. The BFT protocol provides the following properties.

- (**Safety**): Every two honest nodes do not commit different blocks at the same height.
- (**Liveness**): If a transaction is received by an honest node, then the transaction will eventually be included in every honest node’s ledger.

##### Definition 7

(Two-phase Atomic Commit Protocol^9^) This protocol has two phases that are run by a coordinator. During the first voting phase, the nodes tentatively write changes locally, lock resources, and report their status to the coordinator. If the coordinator does not receive *AC* from a node, it assumes that the node’s local writes failed and sends a rollback message to all of the nodes to ensure that any local changes are reversed and that the locks are released. If the coordinator receives *AC* from all of the nodes, it initiates the second commit phase and sends a commit message to all of the nodes so that they can permanently write the changes and unlock resources. In the context of a sharding blockchain, the atomic commit protocol operates on shards (which make the local changes associated with the voting phase via an intra-shard consensus protocol such as the PBFT) rather than on individual nodes.

##### Definition 8

(Availability Certificate^9^) In the 2PC protocol, *AC* is a crucial document generated by each input shard involved in a transaction. These certificates are produced at the end of the first phase of the transaction and indicate each shard’s preliminary reaction to the transaction, namely, whether to accept (preaccept) or reject (preabort) the continuation of the transaction.

### Concrete protocol

**Table 2:** Cross shard leader accountability.

CSLRE: cross-shard leader accountability protocol	
Let $L_{in}$ and $L_{out}$ be the leaders of $shard_{in}$ and $shard_{out}$, and let replica $r_{in}$ and $r_{out}$ be the members of $shard_{in}$ and $shard_{out}$.	
The CSLRE protocol executes the following:	
**i. 2PC-prepare.** Using the BFT algorithm, $r_{in}$ generate *AC* for the *tx* protocol. Then, $L_{in}$ sends *AC* to at least $f+1$ nodes of $shard_{out}$.	
**ii. BFT protocol based on the BB/BA.**	
The generation of $CSLRE-QC$ relies on the BFT protocol.	
**(a) BFT protocol based on the BB.** If $L_{out}$ of $shard_{out}$ has not received *AC* for a relevant input shard $shard_{in}$ or received an error *AC*, $L_{out}$ broadcasts the Cross-Shard Leader Re-election proposal. $r_{out}$ runs the BFT protocol based on the BB to generate $CSLRE-QC$. If $R_{out}$ of $shard_{out}$ still has not received the leader’s proposal information after receiving *AC* for $t0+2\Delta$ (*t*0 is the timestamp of the last accepted *AC*), it runs the view-change protocol.	
**(b) BFT protocol based on the BA.** If $R_{out}$ of $shard_{out}$ has not received *AC* for input shard $shard_{in}$ or received an error *AC*, $R_{out}$ proposes to generate $CSLRE-QC$ to switch $L_{in}$ from among the $R_{out}$.	
$R_{out}$ runs the BFT protocol based on the BA to generate $CSLRE-QC$.	
**iii. CSLRE-QC transfer.** $r_{out}$ sends the generated $CSLRE-QC$ to all $r_{in}$ of $shard_{in}$ and turns on a timer for 2$\Delta$.	
**iv. Leader Re-election.** $r_{in}$, upon receiving $CSLRE-QC$, uses the round-robin approach to switch $L_{in'}$ after determining the authenticity of $CSLRE-QC$. Then, the new leader $L_{in'}$ resends *AC*.	
**v. 2PC-commit.** If $shard_{out}$ re-receives *AC* before 2$\Delta$, the process proceeds to the 2PC-commit as normal; otherwise, it returns to the second step to continue execution.	

Based on the above primitives, we propose our CSLAP protocol. Table 2 provides a complete protocol flow. The following describes the detailed execution process of the agreement through a transaction:

#### 2PC-prepare

 This phase is the first phase of a standard cross-shard transaction (the 2PC-prepare phase). Initially, the client broadcasts the transaction *tx* to all of the shard members. Then, a shard that does not contain the relevant transaction information forwards the *tx* information to the relevant shard and discards the transaction *tx*. The shard retains the transaction *tx* containing the relevant transaction information. Shard members then run the intra-shard consensus algorithm to generate a transaction $AC_i$ and send it to each relevant input and output shard. The input shard $shard_{in_i}$ is used to run the intra-consensus algorithm to generate the transaction $AC_i$. After $T_{BFT}+\Delta$, each relevant shared 1/2 member and leader should have received all $AC_i$ related to *tx*.

#### BFT protocol based on the BB

 As a coordinator shard $shard_{out}$, if the *AC* of all input shard transactions are typically received, then $shard_{out}$ executes the second phase of 2PC. After the members of $shard_{out}$ receive all relevant *AC*, they broadcast the certificate information and start a timer. The timer is set to $t0+2\Delta$, where *t*0 is the timestamp in the last *AC* accepted, indicating the time when the input shards sent *AC*. The shards wait for the leader to take action until it times out, and they perform a view-change protocol to replace the leader. If the $AC_i$ for an input shard is still not received, the leader $L_{out}$ of $shard_{out}$ votes and runs the BFT protocol based on the BB to generate a CSLAP-QC. To submit a proposal, two rounds of voting are needed. $R_{out}$ collects $f+1$ valid votes $<m_{vote}>$ and uses the collected votes to build a CSLAP-QC.

#### BFT protocol based on the BA

 As a coordinator $shard_{out}$, if the *AC* for all input shards is typically received, $shard_{out}$ performs the 2PC-commit. If the *AC* for an input shard is still not received, member $r_{out}$ in $R_{out}$ executes the BFT protocol based on the BA phase. $shard_{out}$ runs the BFT protocol based on the BA to generate a CSLAP-QC. After two rounds of voting, $R_{out}$ collects $f+1$ valid votes $<m_{vote}>$ and uses the collected votes to construct the CSLAP-QC.

#### CSLAP-QC transfer

 After generating the CSLAP-QC in shard $shard_{out}$, $R_{out}$ sends the CSLAP-QC to all members in $shard_{in_i}$ and starts a timer of 2$\Delta$. $R_{in_i}$ first verifies the CSLAP-QC signature; if the verification is passed, the members of $shard_{in_i}$ agree that their leader $L_{in_i}$ is a malicious leader, and $L_{in_i}$ does not provide $AC_{x}$ to other shards. The members of $shard_{in_i}$ initiate a round-robin operation to switch the leader.

#### Leader re-election

 When the current leader $L_{in_i}$ is found to be acting maliciously, nodes $R_{in_i}$ use a round-robin algorithm to elect a new leader $L_{in_{i'}}$. The algorithm uses the latest block in the blockchain as input to select a new leader from all nodes but excludes up to *f* nodes that have exhibited malicious behaviour and have recently served as leaders. The new leader $L_{in_{i'}}$ resends the $AC_i$ information before the block state defaults to show malicious leader behaviour.

#### 2PC-commit

If $shard_{out}$ still does not receive the *AC* for $shard_{in_i}$ before the 2$\Delta$ timer expires, then the leader replaced by $shard_{in_i}$ is considered malicious. The CSLAP continues to execute until $shard_{in_i}$ becomes an honest node. After receiving all of the certificate information, $shard_{out}$ puts the transaction on the chain once the verification is completed. Additionally, it sends the acceptance or rejection information to all of the relevant shards.

### Security analysis

#### Lemma 1

(*Consistency*) If the BA/BB and 2PC of the BFT protocol are consistent, then this agreement is considered consistent.

#### Proof

We assume that *tx* $T\{(I_1,..., I_x)) \rightarrow (O_1,...,O_y) \}$, where $Shard_{out_c}$ is the coordinator shard. The transaction is broadcast by the client to all shards, and the input shard $shard_{in_i}$ needs to generate the corresponding *AC*. The intra-shard BFT consensus is used to generate *AC*, which satisfies consistency for all required certificate stages. At this point, $shard_{in_i}$ locks the relevant $I_i$ and sends *AC* to the coordinator shard. *AC* contains both the preaccept and preabort signals. $Shard_{out_c}$ includes the following cases:

(i) If $Shard_{out_c}$ receives preabort (T), the transaction is considered invalid. The coordinator shards execute BFT consensus and send abort(T) to all related shards, indicating that the transaction has failed. The input shard unlocks $I_i$. During this process, message broadcasts are sent to all shard members, ensuring that the members are informed. If a leader fails to send messages honestly, Shard members can still use the round-robin protocol to replace the leader, which also satisfies consistency. Therefore, this process satisfies consistency.
(ii) During the 2PC process, two conflicting transactions cannot be submitted simultaneously, ensuring that this protocol is immune to double-spending attacks. When $Shard_{out_c}$ has received enough evidence indicating preaccept(T) for each shard, the transaction is considered valid. $Shard_{out_c}$ reaches a consensus on the transaction through the BFT and broadcasts the message to all shards. The CSLAP ensures that the message is broadcast to all members of all shards, ensuring that all shards receive the message. At this point, all of the input and output shards reach a consensus regarding the final outcome of the transaction, satisfying consistency.
(iii) If the leader of $shard_{in_i}$ is compromised and fails to send *AC* to some members, $Shard_{out_c}$ runs the BFT to generate the CSLAP-QC, which is transmitted to its sender within the specified time frame. After receiving the CSLAP-QC, the input shard members save the current state for the round-robin process, ensuring that the current state in the blockchain is consistent with the pre-round-robin state. The new leader rebroadcasts *AC*. At this point, $shard_{in_i}$ rebroadcasts, while $Shard_{out_c}$ has not received all the *AC*, leading to a consistent waiting state for the transaction *tx*. The round-robin protocol does not affect the consistency property. Hence, the sharding protocol does not conflict with public prefixes and shards.

When $Shard_{out_c}$ sends the CSLAP-QC, a 2$\Delta$ timer is initiated. If the timer expires without receiving *AC* from $shard_{in_i}$, the CSLAP protocol is re-executed until the *AC* from $shard_{in_i}$ is received. During this process, all shards are either consistent or in a waiting state, meeting the consistency requirement.

Thus, the sharding system can maintain transaction consistency, proving the correctness of the protocol. $\square$

#### Lemma 2

(*Liveness*) For any valid transaction *tx* submitted by a client, after a certain period, all relevant shards finally decide to execute accept(T) or abort(T).

#### Proof

Let us assume that the start moment occurs when a client’s transaction request is sent to all input shards. The liveness parameters are related to the parameters of the BFT protocol. For the input to a transaction, the input shard takes $T_{BFT}$ time to generate *AC* and $\Delta$ time to send *AC* to the relevant shard. During the 2PC-commit phase, $shard_{out}$ also needs $T_{BFT}$ time to commit the transaction. It takes $T_{BFT}+\Delta$ to provide feedback to the input shard and reach full agreement. Therefore, when the relevant leaders are honest and optimistic, the transaction confirmation parameter $T_{liveness}$ can be calculated as $3T_{BFT}+3\Delta$.

There are two cases of proof of liveness.

**Part I**: The leaders of all relevant shards in a transaction are honest. First, assuming that the leaders of all relevant shards for a transaction *tx* are honest, the input shard generates the *AC* of $tx^i$ through the BFT algorithm. Then, the input shard leader sends *AC* to the relevant shard. In $shard_{out}$, after receiving all *AC* related to *tx* in $shard_{out}$, $shard_{out}$ runs the intra-shard consensus algorithm and submits *tx* to the block.

**Part II**: The leaders of some shards are dishonest. The second case occurs when some input shard leaders are malicious. Malicious leaders can censor transactions in three ways:

(i) After receiving the *tx* value from the client during the 2PC-prepare phase, the leader of the input shard is withheld.
(ii) The input shard generates *AC* through the BFT protocol, but the leader of the input shard does not send *AC* to $shard_{out}$.
(iii) The input shard generates *AC* through the BFT protocol, but the leader of the input shard sends a conflicting *AC*.
  Case i: The member of the input shard does not receive the 2PC preparation proposal within the $\Delta$ time of receiving the transaction information *tx* and initiates the active view-change mechanism.
  Case ii/iii: After $shard_{out}$ receives *tx* and waits for $T_{BFT}+\Delta$ time, the BB/BA protocol generates the CSLAP-QC and sends it to the input shard. The input shard executes the round-robin protocol to replace the leader.
  In Case ii/iii, considering the best case, the input shard is replaced with an honest node after executing the round-robin protocol once, and *AC* is sent normally; then, the protocol execution time is $T_{BFT}+2\Delta$. The transaction confirmation parameter is $T_{liveness}=4T_{BFT}+4\Delta$. In the worst case, if the input shard executes *k*($k<=f-1$) round-robin iterations before replacing it with an honest node, the shard needs to wait for $2\Delta$ every time *CSLAP* is re-executed. Therefore, the protocol execution time is $k(T_{BFT}+2\Delta )+2\Delta +3T_{BFT}$, and the transaction confirmation parameter is $T_{w-liveness}=(k+3)T_{BFT}+2(k+1)\Delta$. If an adversary is able to disrupt the liveness of *CSLAP*, it must either disrupt the voting power in the network by more than 1/2 or disrupt the termination of the BFT protocol based on the BB/BA, which has only negligible probability. Thus, the sharding system can maintain transaction liveness, proving the protocols correctness.

$$\square$$

#### Theorem 1

In the synchronous network model, assuming that multiple malicious behaviours exhibited by different shard leaders are detected by different shards in the shard blockchain, the leaders of all malicious shards are still replaced in $T_{liveness}$.

#### Proof

This protocol allows parallel processing of malicious leaders behaviour, assuming that in a transaction, the leader of an honest shard detects the malicious behaviour of one or more leaders of the related shards. Since generating a CSLAP-QC certificate does not involve proposing blocks or generating any sequence of actions, the leader can simultaneously propose the BFT for malicious behaviour on multiple shards. After the certificate is generated, the shard members send the certificate to the relevant shards. Therefore, regardless of whether several leaders exhibit malicious behaviour, they will be the current malicious leaders within $T_{liveness}$. $\square$

#### Theorem 2

Starting with sending *AC* *t*0, the coordinator shard members detect the malicious behaviour of this shard within the maximum *t*0+$2\Delta$ time and initiate a view change to switch and replace the current leader.

#### Proof

Assuming a transaction, after generating *AC*, the time when the shard leader sends *AC* is *t*0, and the leader sends *AC* to the $f+1$ members of the coordinator shard at the same time. The time when the members start receiving *AC* is *t*. *AC* contains the *t*0 timestamp. The time at which the shared leader receives *AC* is $t'$, $t0$ ($t,t'$) $t0$+$\Delta$. Assuming that the shard leader is honest, the shard members receive *AC* within $t'$+$\Delta$ and *t*0+$\Delta <t'$+$\Delta <t0$+$2\Delta$. Assuming that the coordinator shard leader is malicious, the coordinator shard members will not receive the *prepare* information from the coordinator shard leader within $t'$+$\Delta$. If $t$ $t'$, then $t'$+$\Delta <t$+$\Delta$ $<t0$+$2\Delta$; if $t<t'$, then *t*+$\Delta <t'$+$\Delta <t0$+$2\Delta$. $\square$

### Efficiency analysis

#### Time complexity

From the moment malicious behaviour is detected, if one round-robin is executed to replace the input shard with an honest node, then the protocol execution time is $T_{BFT} + \delta$, where $\delta$ represents the actual transmission delay in the network. In the worst-case scenario, if the input shard needs a total of *k* rounds of polling (where $k <= f - 1$) before switching to an honest leader, and if each time the CSLAP is re-executed, it must wait for the maximum message round-trip delay of $2\Delta$, then the time for *k* rounds of polling is $k \times (T_{BFT} + 2\Delta )$.

The final successful replacement takes $T_{BFT} + 2\delta$. Hence, the total execution time for the protocol is $(k + 1) \times T_{BFT} + 2k \times \Delta + \delta$.

Regarding the time complexity of the BFT, considering the PBFT as an example, its asymptotic time complexity is $O(n^2 \times \Delta )$, where *n* is the number of members in a single shard. Therefore, the execution time complexity can be represented as $O((k + 1) \times (n^2 \times \Delta ) + 2k \times \Delta + \delta )$. Given that $k <= f - 1$ and $f <= \frac{n - 1}{2}$, we have the following:

1 $$\begin{aligned} O((k + 1) \times (n^2 \times \Delta ) + 2k \times \Delta + 2\delta )<= O(f \times (n^2 \times \Delta ) + 2k \times \Delta + 2\delta ) <= O\left( \frac{n - 1}{2} \times (n^2 \times \Delta ) + 2k \times \Delta + \delta \right) . \end{aligned}$$

Since $2\delta$ is constant and $2k \times \Delta$ is smaller than $(\frac{n - 1}{2}) \times (n^2 \times \Delta )$, the time complexity can be understood as $O((\frac{n - 1}{2}) \times (n^2 \times \Delta ))$.

In the best-case scenario, in which only one swap needs to be replaced with an honest leader, the time complexity is $O(n^2 \times \Delta )$. In the worst-case scenario, in which $f - 1$ swaps are needed, the time complexity is $O((\frac{n - 1}{2}) \times (n^2 \times \Delta ))$. Thus, the overall asymptotic time complexity is $O(n^3 \times \Delta )$.

#### Communication complexity

When the system executes the CSLAP, the honest leader needs to broadcast, and the broadcast is one-to-many; therefore, the communication complexity is *n*, which is the number of members in a shard. When no message is received, the communication complexity of executing a BFT round (using the PBFT as an example) is $O(n^2)$. Then, the process of regenerating the certificate and sending it is a multicast process; thus, there are n members in the fragment broadcast, and the communication complexity is $O(n^2)$. Finally, if the other shards are polled, the communication complexity is 1. If k rounds are performed, then the total communication complexity is $k(O(n^2+n^2+1)=O(k*n^2)$. As described in Sect. "Time complexity", the best communication complexity is $O(n^2)$, and the worst communication complexity is $O(n^3)$. Therefore, the asymptotic time complexity is $O(n^3)$.

### Performance analysis

Table 3 shows a comparison of the protocols. Identifying malicious leadership represents whether a leader with malicious behaviour can be accurately identified for each cross-transaction censorship attack.

**Figure 2:**
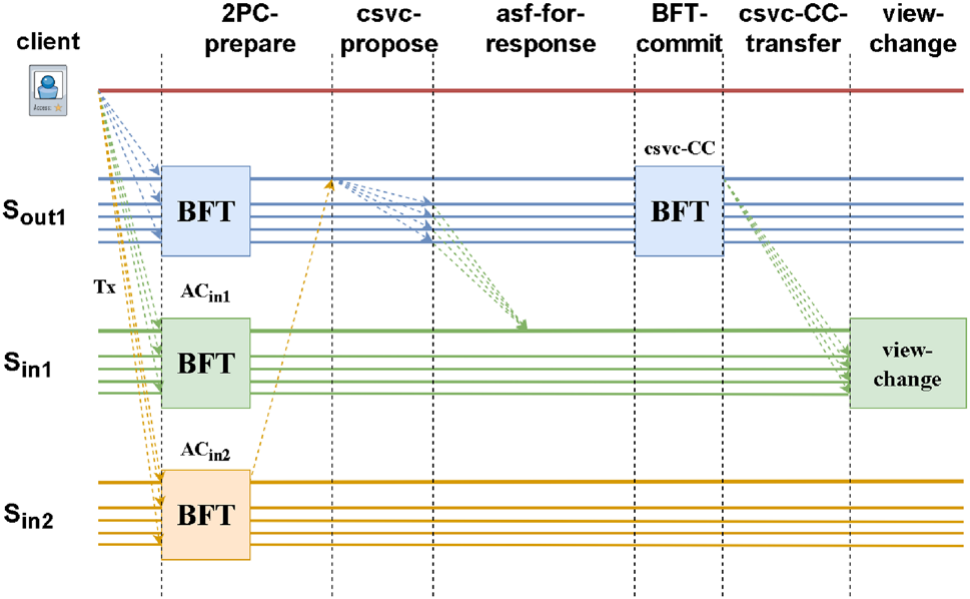
Cross-shard view-change protocol.

**Figure 3:**
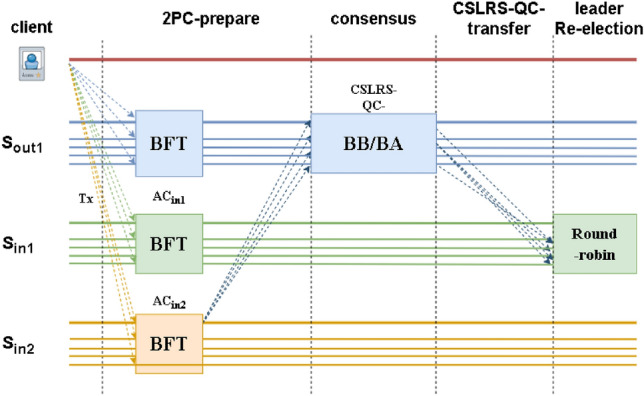
Cross shard leader accountability protocol.

**Figure 4:**
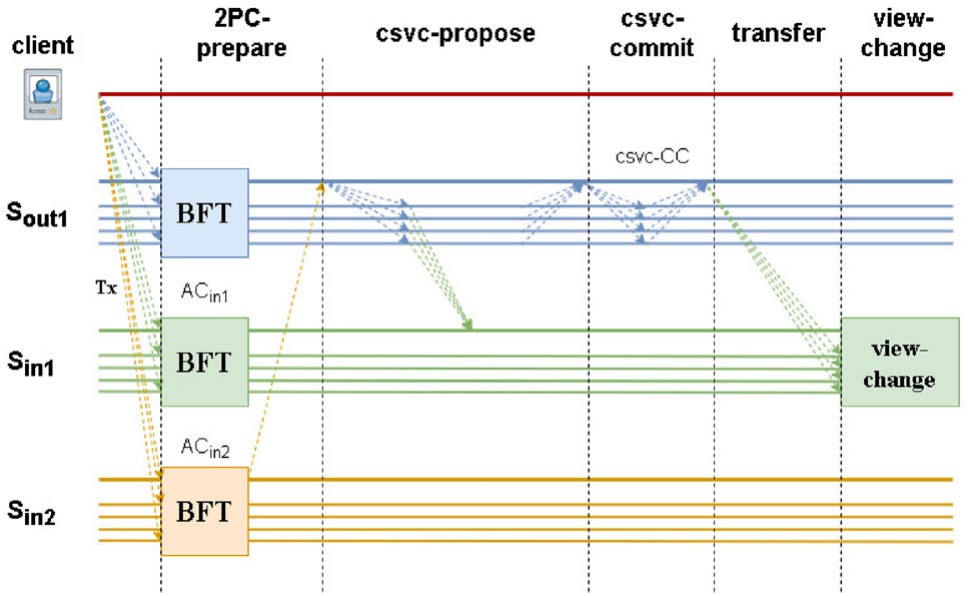
Flexible sharding cross-shard view-change protocol.

**Table 3:** Comparison among the CSLAP and other protocols.

System	Network model	Adversary model	Identify malicious leadership	Time
CSVC	Sync	${\frac{1}{2}}$	$\times$	$T_{\textrm{BFT}} + 2\delta + 2\Delta$
FS_CSVC	Partial sync	${\frac{1}{3}}$	$\times$	$T_{\textrm{BFT}} + \delta + 2\Delta$
CSLAP	Sync	${\frac{1}{2}}$	√	$T_{BFT}+\delta$

Figure 2 shows the implementation process of the CSVC protocol, Fig. 3 is the execution process of the CSLAP, and Fig. 4 is the execution process of FS_CSVC. (We only focus on the security and consistency of transactions between shards and do not consider the transaction consensus process within shards.) In the CSVC, after leader $L_{out_1}$ of $S_{out_1}$ discovers the malicious behaviour of $S_{in_1}$, it notifies the members first, and then, the members ask $L_{in_1}$ of $S_{in_1}$. If $L_{in_1}$ provides a message to the inquiring member of $S_{out_1}$, then the member broadcasts *AC* in $S_{out_1}$, and the shard leader continues to process *tx* in the normal 2PC protocol; if $R_{out_1}$ does not receive *AC* from $L_{in_1}$ within $2\Delta$, it provides $L_{out_1}$ with an agreement message, which is included in the BFT-commit phase. $L_{out_1}$ then runs the BFT algorithm to generate a cross-shard view change (CSVC-CC). Next, $L_{out_1}$ sends the CSVC-CC to $f+1$ members of $S_{in1}$, and $S_{in_1}$ members attempt to switch after verifying the certificate. We regard the two consensus rounds as $T_{BFT}$ and use $\delta$ to denote the real-time delay when a node sends information to other nodes. Therefore, leader replacement requires $T_{BFT} +2\delta +2\Delta$.

$L_{out_1}$ sends vcs-prepare to the shard first, and $R_{out_1}$ receives the vcs-prepare request $L_{in_1}$ query. If no message is received, the shard signs vcs-prepare. $L_{out_1}$ then constructs csv-commit after receiving $2f+1$ signatures and generates $csvc-CC$ after collecting $2f+1$, and the message is broadcast to all members of $S_{in_1}$ to change the view. The time required for a leadership change is $T_{BFT}+\delta +2\Delta$.

FS_CSVC adopts two rounds of BFT consensus. $L_{out_1}$ first sends CSVC-PREPARE to the inside of the shard, and $R_{out_1}$ receives CSVC-PREPARE to ask $L_{in_1}$ queries and signs CSVC-PREPARE if no message is received. $L_{out_1}$ then constructs CSVC-COMMIT after receiving the $2f+1$ signature and generates the $csvc-CC$ information after collecting $2f+1$. Then, the information is sent to all members of $S_{in_1}$ for a view change. The time required to replace the leader is $T_{BFT}+\delta +2\Delta$.

The CSLAP protocol uses $S_{in_1}$ to send certificate information to at least $f+1$ $S_{out_1}$ to ensure that $L_{in_1}$’s malicious behaviour can be determined without request, and $S_{out_1}$ is sent to all members of $S_{in_1}$ after the CSLAP-QC is generated.

At this time, all members have reached an agreement on the malicious behaviour of the leader so that the leader can be replaced directly through the round-robin protocol, and there is no need to change the view and internal state through a view change. Since the round-robin protocol directly replaces its own information, the time consumed is negligible. In this context, regardless of whether the BFT protocol is based on the BB or BA, the runtime is uniformly considered $T_{BFT}$. The time required for the protocol to replace a malicious leader is $T_{BFT}+\delta$. Thus, $T_{CSVC}>T_{FS\_CSVC}>T_{CSLAP}$.

## Implementation & evaluation

We seek to answer the following questions through this evaluation:

- What is the system’s performance for two different intra-shard BFT protocols and three cross-shard lead conversion protocols under conditions of varying transaction sizes, maximum delays, and shard counts? ("Performance evaluation of the intra-shard and cross-shard lead conversion protocols")
- What is the system’s performance when running the conventional two-phase commit protocol under conditions with different numbers of transaction rounds? ("Performance evaluation of the two-phase commit protocol")
- What is the system’s performance when, for example, executing multiple rounds of cross-shard transactions in the presence of a malicious leader in one of the input shards? ("Performance evaluation in complex networks")

### Implementation details and methodology

#### Experimental setup

We deployed our implementation on a cloud instance from the Alibaba Cloud^29^. We used an ecs.g7.8xlarge instance (32 vCPUs, 64 GB of memory, and 25 GB/s network bandwidth) in our Hangzhou data centre^30^. The instance operates on a dedicated network with a network latency of no more than 20 ms, runs Windows 11 as the operating system, and uses 100 GB of SSD storage. In our experiment, the main metrics were latency (quantified in seconds) and throughput (quantified in bytes processed per second).

#### Implementation

**Figure 5:**
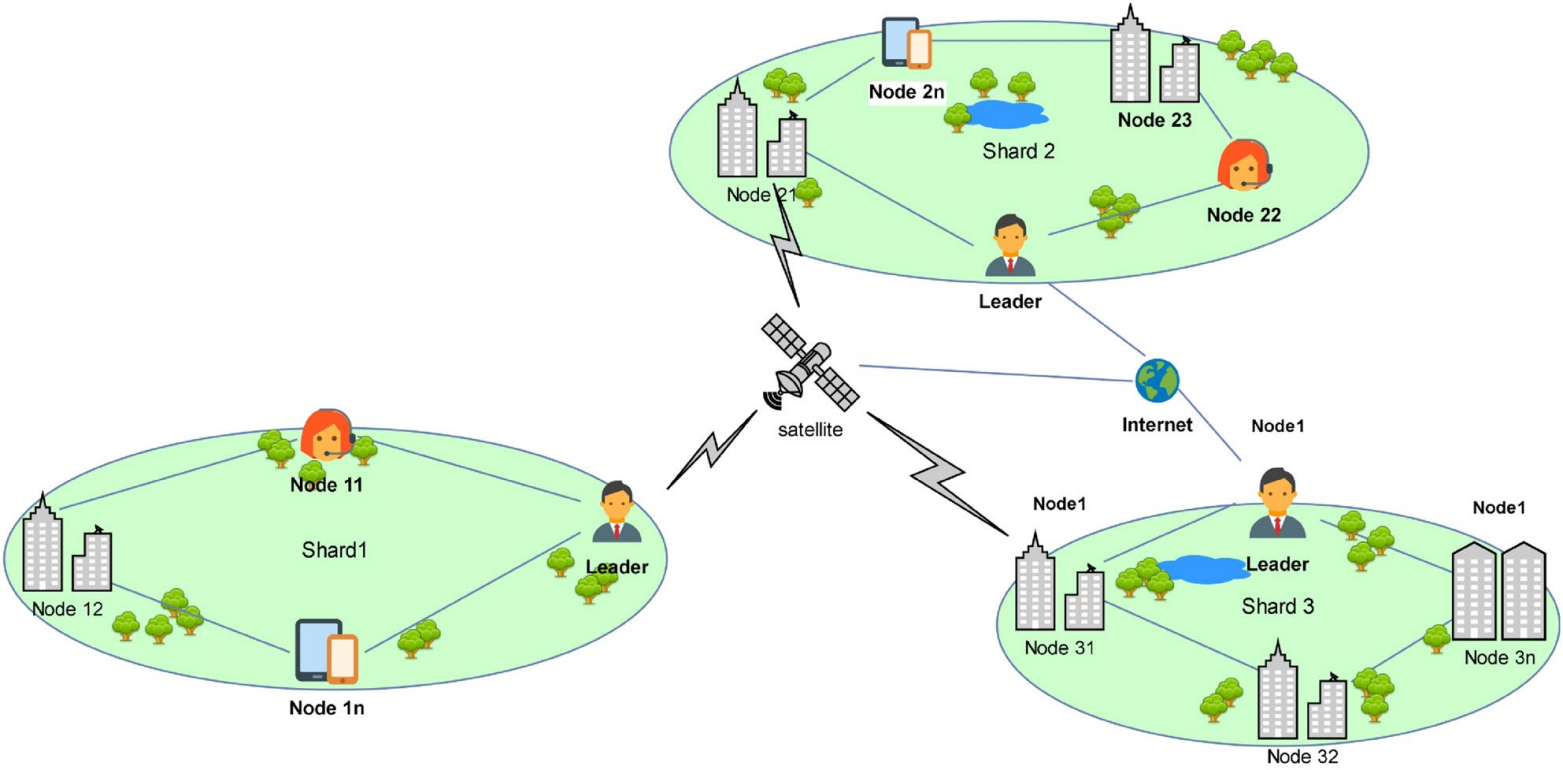
Network topology structure.

 We implemented a two-stage atomic commit model under the standard synchronous model. Shards mainly use BFTs based on the BB prototype to synchronize HotStuff (SYNC)^31^ and reputation-based state machine replication (RBSMR)^26^ for certificate information generation and final transaction confirmation. We modified the core logic of SYNC and the RBSMR by adding a cross-shard transaction model to ensure that messages sent from other shards are recognized and processed within the chip. Shards use a 2PC to interact with each other. The CSVC, FS_CSVC and CSLAP are three cross-shard lead conversion protocols. For different cases, we use the following abbreviations: When the intra-shard consensus is SYNC, there are no cross-shard lead conversion protocols between shards. In short, for SYNC, when the CSVC is used in the 2PC, it is called SYNC-CSVC; when using FS_CSVC, it is called SYNC-FS; and when the CSLAP is used, it is called SYNC-CSLAP. The same is true when the intra-shard consensus is the RBSMR. We used fmt to output content and log to record log information, and we utilized net/http to simulate each of the different nodes. Additionally, we used the sync package to achieve synchronization among multiple goroutines. Moreover, we have reused the intra-shard consensus operations from SYNC and the RBSMR. In our implementation, the transactions proposed by customers are all cross-shard transactions and are specific to $T\{(I_1, I_2)\rightarrow (O_1, O_2, O_3)\}$. $Shard_{out_3}$ is the coordinator shard by default. Other shards submit *AC* to $shard_{out_3}$. The model only processes the next transaction after executing one transaction. All throughput and latency results are measured from clients of separate processes that run separately on the same virtual machine as the shard members. We ensured that the performance of sharding is not limited by the lack of transactions proposed by the client. The network topology of the system is shown in Fig. 5.

### Experiment

#### Performance evaluation of the intra-shard and cross-shard lead conversion protocols

**Figure 6:**
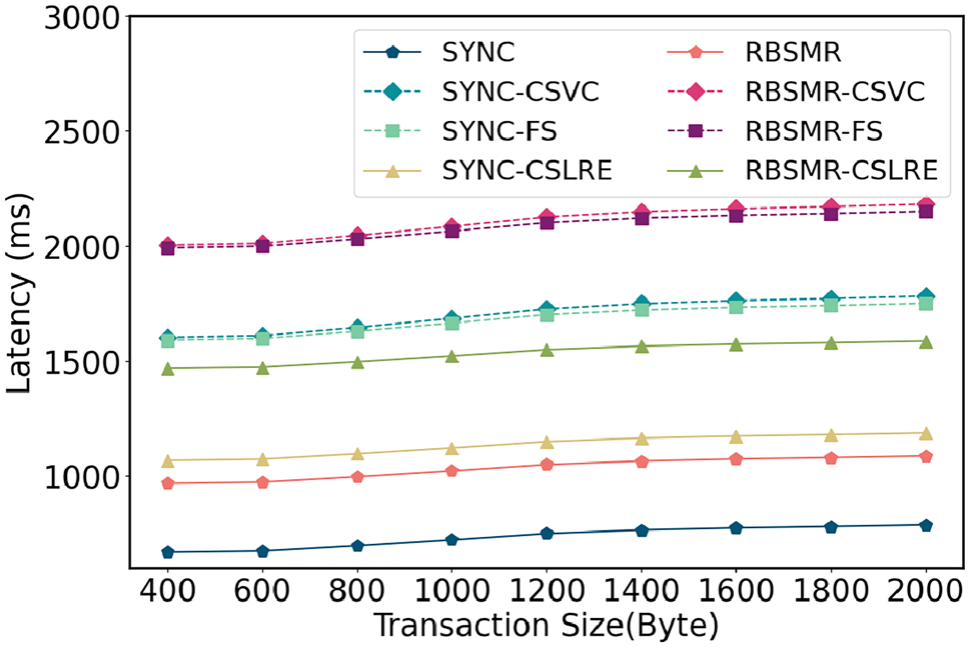
Latency comparison of different transaction sizes.

We first evaluated the baseline latency of a single-round 2PC protocol with fault tolerance ($f = 1$) in a synchronous setting with $\Delta = 100$ ms. The number of members in each shard *m* was set to 4. Initially, we fixed the number of shard members and varied the transaction sizes, considering nine different transaction sizes ranging from 400 to 2000 bytes. For each transaction, we compared the latency of two distinct intra-shard consensus protocols, a standard two-phase commit protocol and cross-shard lead conversion protocols in the presence of a malicious leader. Each data point represents the latency from when the client broadcasts the inter-shard transaction to when the transaction is confirmed. After the client broadcasts the transaction, different shards reach a consensus, lock the transaction, and provide *AC* to the coordinating shard. As shown in Fig. 6, under honest shard conditions, the latencies for SYNC and the RBSMR were 668 ms and 956 ms, respectively; with a malicious leader, the latencies for SYNC-CSVC and SYNC-FS were approximately 1600 ms, and for RBSMR-CSVC and RBSMR-FS, they were nearly 2000 ms, with this protocol exhibiting latencies of approximately 1000 ms and 1500 ms, respectively. The presence of a malicious leader significantly increases the execution time. As the transaction size increases, the latency tends to increase; this phenomenon mainly due to the increase in packet size and the extended data transmission time.

**Figure 7:**
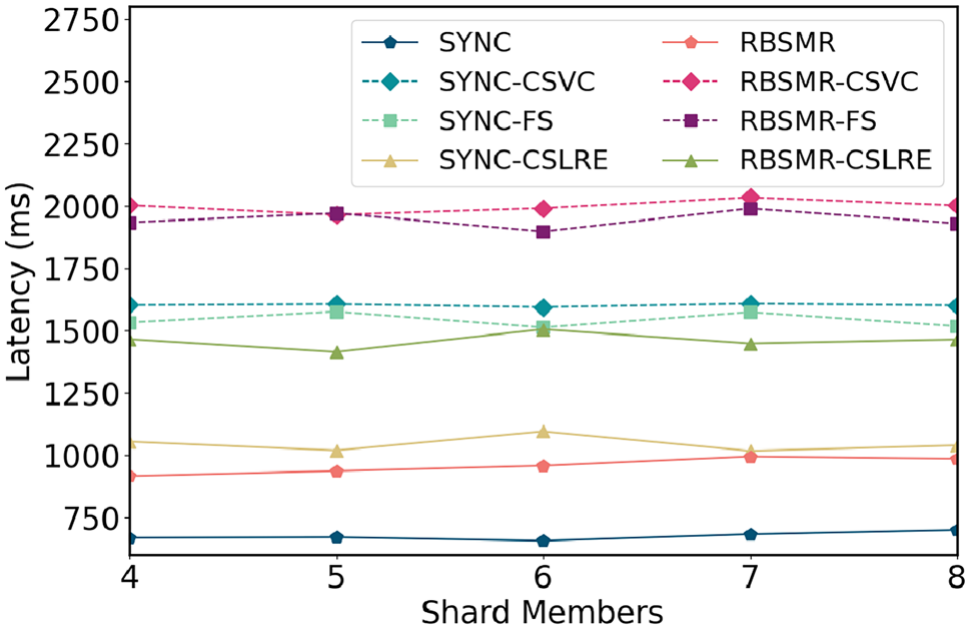
Latency comparison of different numbers of shard members.

**Figure 8:**
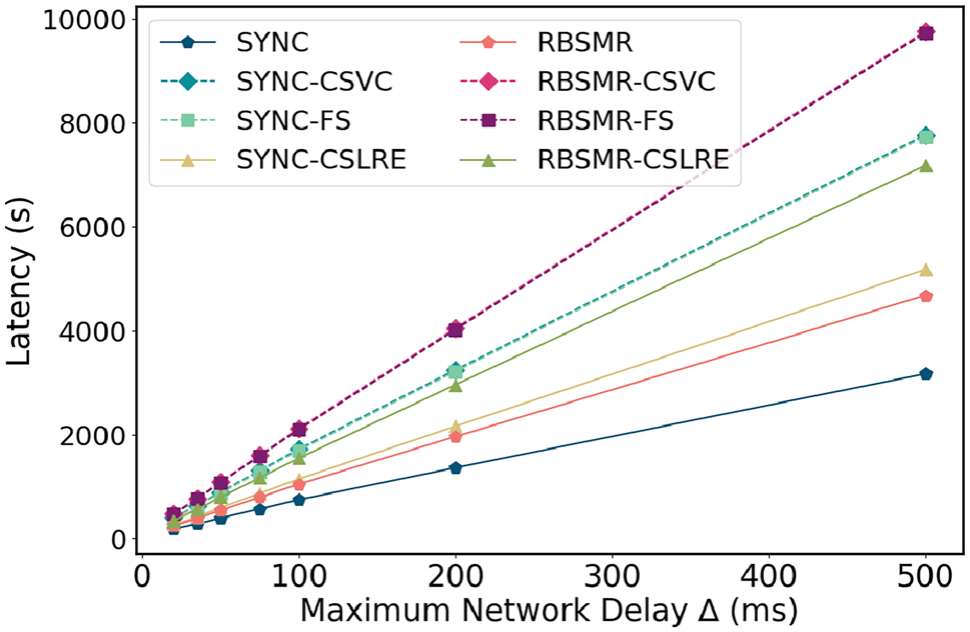
Latency comparison of different MAX($\Delta$) values.

Next, we fixed the transaction size and maximum transmission delay and varied the number of shards from 4 to 8. As depicted in Fig. 7, with changes in the number of shard members, since both internal consensus protocols are synchronous network models, the time taken typically fluctuates with the number of nodes, where system fluctuations are essentially network fluctuations.

Furthermore, we fixed other variables and set the maximum network delay to 20, 35, 50, 75, 100, 200, and 500 ms. As illustrated in Fig. 8, at $\Delta = 20$ ms, the latency for SYNC was 188 ms, and the latencies for SYNC-CSVC, SYNC-FS, and SYNC-CSLAP were 400, 380, and 268 ms, respectively. At $\Delta = 500$ ms, the latencies for these protocols were 3174 ms for SYNC and 7761, 7600, and 5174 ms for SYNC-CSVC, SYNC-FS, and SYNC-CSLAP, respectively. Their multiples increased from 2.12, 2.02, and 1.42 to 2.44, 2.39, and 1.63, respectively. These results demonstrate that the smaller $\Delta$ is, the shorter the execution time of the protocols, the lower the impact of $\Delta$, and the greater the impact of $\delta$. As $\Delta$ increases, both SYNC and the BFT-SMR have a fixed timer cost, leading to a significant increase in network latency.

**Figure 9:**
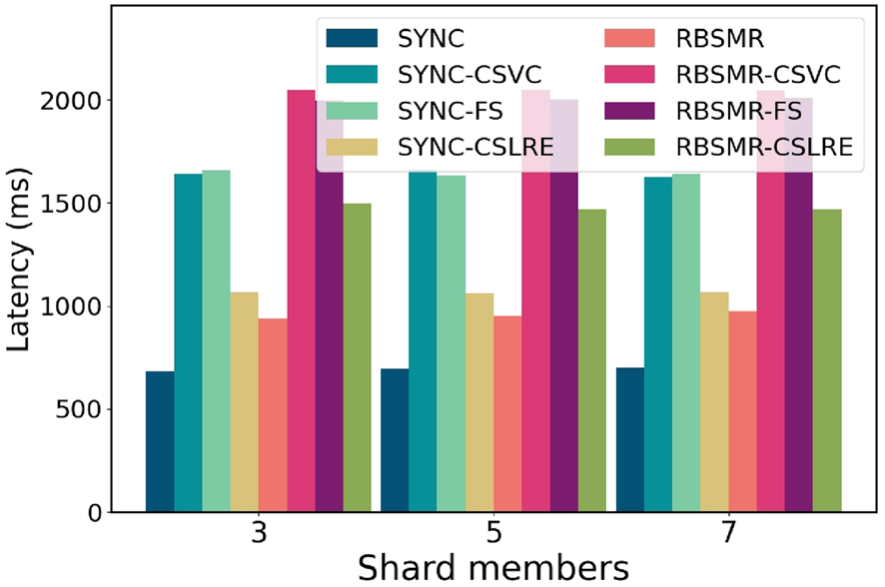
Time delay of different shard members.

**Figure 10:**
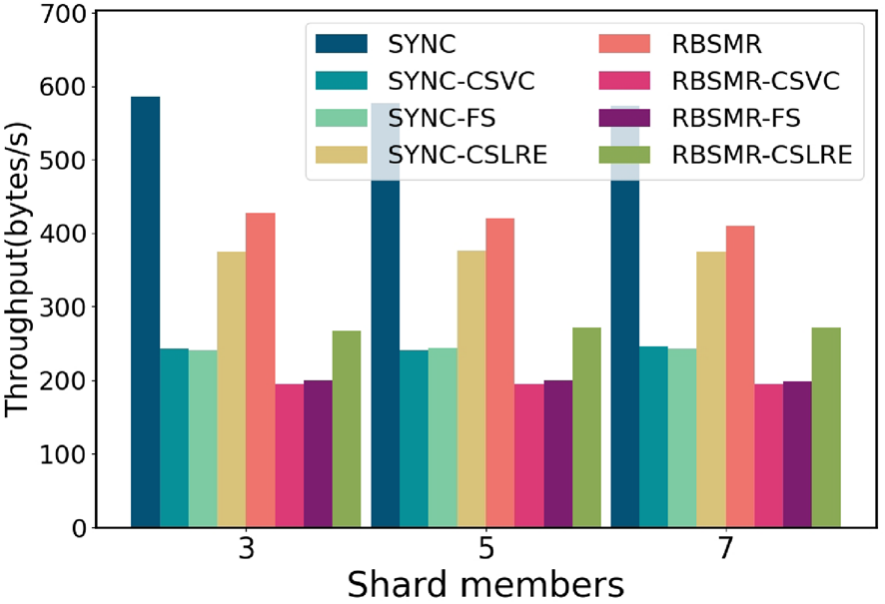
Throughputs of different shard members.

Finally, we present the latency and throughput variations of the system during a round of transactions when the number of shards is 3, 5, and 7. The fixed transaction size in the system is 400 bytes, with $\Delta$ set to 100 milliseconds, and each shard has 4 members. As shown in Fig. 9, when the number of shards is 3, the system’s latency is consistent with the above figure. Moreover, as the number of shards increases, the latency fluctuation remains within the 10 ms range due to the synchronous network model, in which the system exhibits fixed latency behaviour during execution. Fig. 10 shows that when the number of shards is 3, the throughput of SYNC is 580 bytes/s, while those of SYNC-CSVC and SYNC-FS are 243 and 241 bytes/s, respectively. SYNC-CSLAP achieves 375 bytes/s. The throughput of the RBSMR is 426.744 bytes/second, while those of the RBSMR-CSVC, RBSMR-FS, and RBSMR-CSLAP are 195, 200, and 267 bytes/s, respectively. We observe that the throughput of the RBSMR-CSLAP is 50% and 35% higher than that of the other two protocols. As the number of shards increases, there are no changes in latency or throughput, which is attributed to the synchronous network model since it causes the system to exhibit consistent latency behaviour during execution.

In summary, in our system environment, the regular latency for a 2PC based on two intra-shard consensus protocols ranges between 660 and 960 ms, and protocols requiring leader changes result in greater latencies. The execution time for both the CSVC and FS_CSVC is three times that of the standard 2PC protocol. However, for regular submissions, the protocol requires only 1.5 times the latency. Compared to the CSVC and FS-CSVC, this protocol offers a performance improvement of approximately 50%.

#### Performance evaluation of the two-phase commit protocol

**Figure 11:**
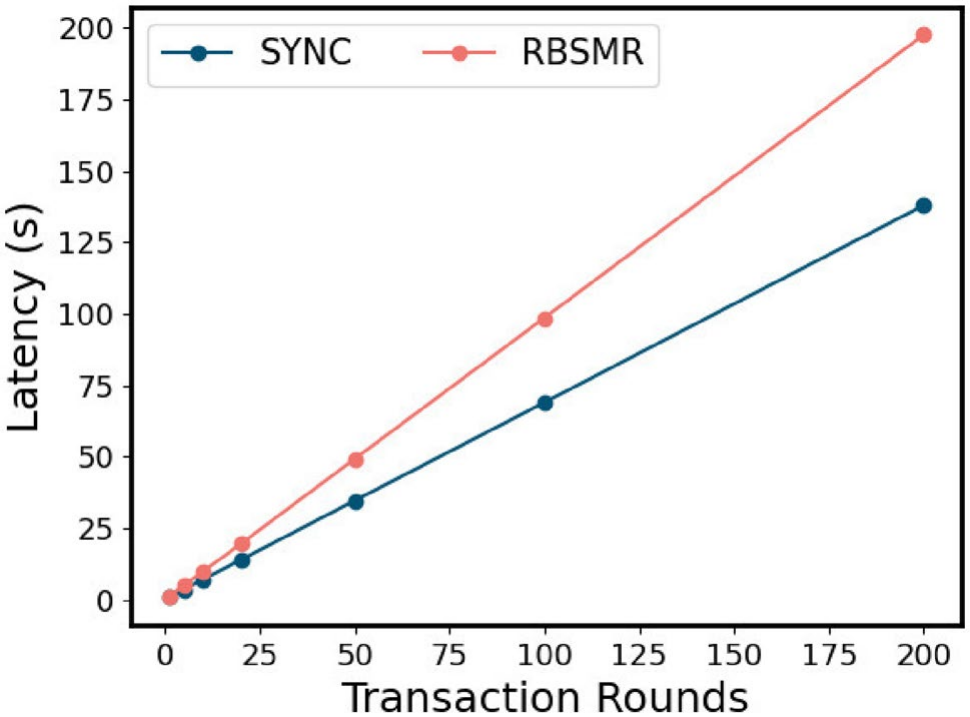
Delay of the two-phase atom submission transaction argument.

**Figure 12:**
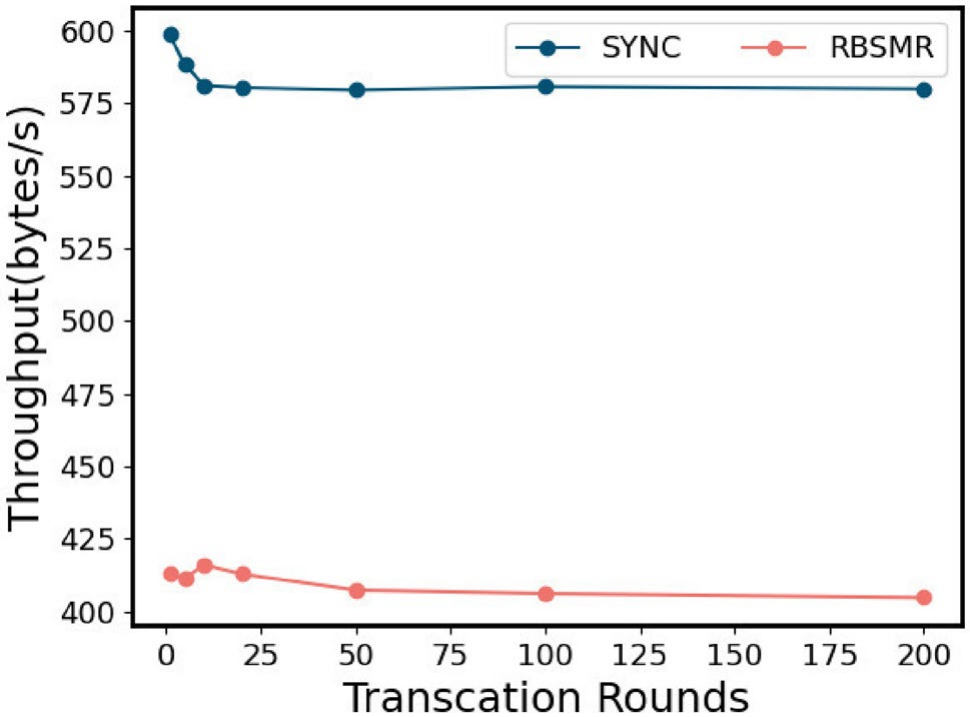
Throughput of the two-phase atomic submission transaction argument.

Next, we evaluated the latency consumed by a normal 2PC. We believe that after executing a cross-shard transaction, we can continue to execute the next transaction. All nodes in the system are honest nodes and implement the agreement as soon as they receive the message. Therefore, this test assesses the time delay and throughput of the system in the honest case. Theoretically, a normal round of two-stage atomic commits needs to go through $3T_{BFT}+3\delta$. Since $\Delta$ is set to 100 ms, the transaction size is 400. As shown in Sect. "Performance evaluation of the intra-shard and cross-shard lead conversion protocols", the SYNC latency is 660 ms, while the RBSMR latency is 970 ms (Fig. 9). Therefore, the sync throughput is 600 bytes/s, and the RBSMR is 410 bytes/s. We began to record the delay and throughput of the number of rounds executed (1, 5, 10, 20, 50, 100, and 200).Fig. 11 shows that the delay consumed by the system execution process is relatively stable overall, and it has little effect on factors such as network delay. It can be seen from Fig. 12 that the initial throughput was close to the throughput limit, but with the operation of the system, the network fluctuated. As a result, the data remained at approximately 575 bytes/s and 400 bytes/s.

#### Performance evaluation in complex networks

**Figure 13:**
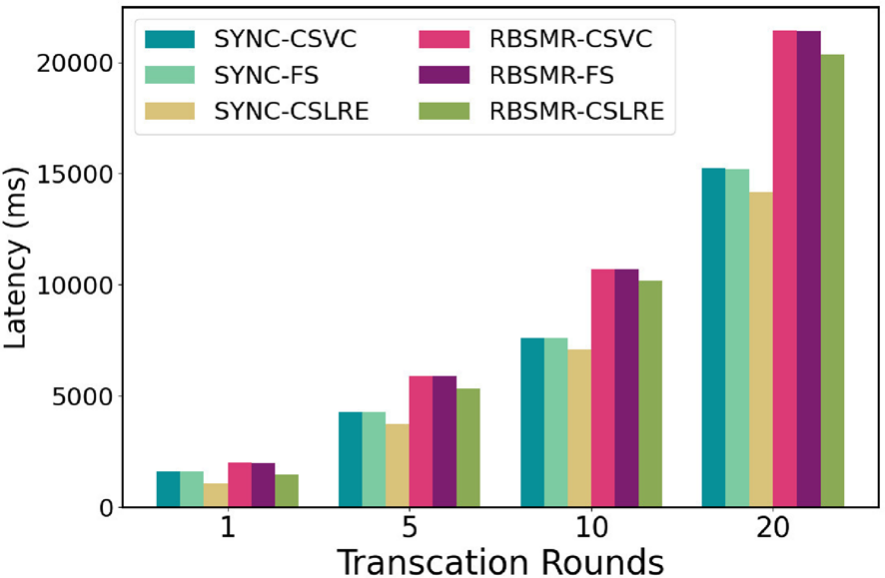
Time delay of different trading rounds.

**Figure 14:**
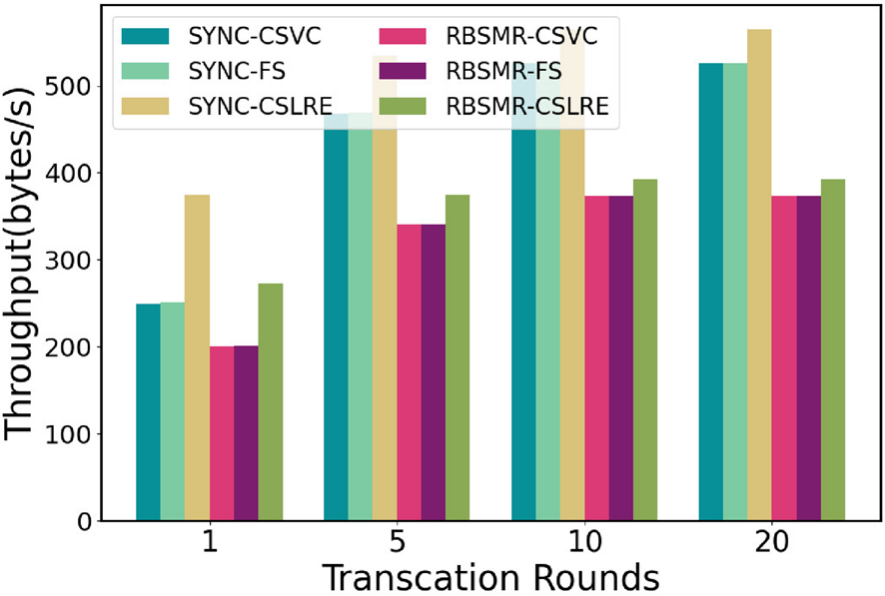
Throughputs of different trading rounds.

Sections "Performance evaluation of the intra-shard and cross-shard lead conversion protocols" and "Performance evaluation of the two-phase commit protocol" illustrate the execution processes of the 2PC protocol under basic conditions. Next, we aim to measure the latency and throughput of the system when executing cross-shard lead conversion protocols in the presence of malicious nodes. We set $Shard_{in_1}$ to contain 20 members, 9 of which were malicious. The other shards remained honest. These malicious nodes, once elected as leaders, may engage in withholding behaviours. We then evaluated the system’s throughput and latency over 5, 10, and 20 rounds, as depicted in Figs. 13 and 14.

From a latency perspective, the system maintains the same delay for one round, as shown in Sect. "Performance evaluation of the intra-shard and cross-shard lead conversion protocols", but the overall ratio of latency across 5 rounds slowly approaches similarity. For instance, SYNC-CSVC exhibited a latency of 1600 ms in one round, while SYNC-CSLAP exhibited a latency of 100 ms. After 20 rounds, the total elapsed time was 15234 ms and 14165 ms, respectively, with the performance improvement decreasing from 1.5 to approximately 1.07 times. Similar patterns are observed with other protocols.

In terms of the throughput, in a single round, SYNC-CSVC, SYNC-FS, SYNC-CSLAP, the RBSMR-CSVC, the RBSMR-FS, and the RBSMR-CSLAP have throughputs of 249, 251, 374, 199, 200, and 272 byte/s, respectively. After 20 rounds, these values increase to 525, 525, 564, 373, 373, and 392 bytes/s, respectively.

These results indicate that malicious behaviour affects the overall system latency and throughput. However, as the number of rounds increases, the throughput gradually increases and approaches the performance levels of the previous honest experiments. This improvement occurs because malicious leaders are identified and replaced promptly, with the only performance loss occurring during the replacement process. In protocols such as SYNC and the RBSMR, nodes identified with malicious behaviour are recorded and prevented from being re-elected as leaders. Our protocol demonstrates faster throughput recovery in the presence of malicious nodes; in scenarios with one round of malicious activity, the throughput is approximately 50% higher than that of the other two protocols, demonstrating less impact from malicious leadership.

## Conclusion

We have defined a cross-shard transaction censorship attack, which threatens the liveness of shard-led cross-shard consensus protocols. This type of attack involves a malicious leader acting honestly within a shard but concealing information between shards. Additionally, the complexity of communication protocols between shards plays a significant role in the efficiency of consensus protocols. To address these issues, we propose the CSLAP, a low-latency consensus protocol designed to resist cross-shard transaction censorship.

We implemented a prototype of the CSLAP and compared it with previous protocols, and the results show that the CSLAP not only resists cross-shard transaction censorship attacks but also has lower communication latency than other protocols. This design is intended to address malicious behaviour in cross-shard consensus, primarily ensuring trusted interactions between shards. Thus, the protocol can be applied to protocols using atomic commits for cross-shard communication.

This paper mainly focuses on identifying and replacing malicious leaders in cross-shard transactions, and it uses the synchronous network model. The message arrives at the maximum network propagation delay, and the network conditions in real life may not be ideal. Therefore, in the nonsynchronous network model, the honest node may be switched due to the timing of resending cross-shard leader-re-election certificates. Second, the BFT-SMR based on the BA cannot meet the safety and activity requirements when the majority of nodes are not honest^32^. The BB-based BFT-SMR cannot satisfy safety and activity requirements with constant rounds^33^. Although we have significantly reduced the time needed to replace malicious leaders, this process may require multiple broadcasts and multicasts, increasing the complexity of inter-shard communication. Additionally, the leader replacement process might continue until an honest node is found. In rare cases, there might be consecutive malicious nodes, leading to a significant increase in delay.

In terms of potential impact and applications, our work provides new insights and solutions for the design and implementation of sharding blockchain systems. Our methods and findings can guide the design and development of future sharding blockchain systems, enhancing their security.

## Future work

The experiments conducted in this paper focus on simulating the process of a single protocol. In future work, we aim to deploy this process in a complete sharding blockchain system or apply other mechanisms to ensure correct transaction confirmation even in the presence of multiple consecutive malicious leaders. We also plan to adapt the CSLAP protocol to a partially synchronous network model, enhancing its stability and reliability under varying network conditions.

Because this study primarily explores communication among malicious leaders and since the protocol only adds some additional measures during inter-shard communication without altering the fundamental process of cross-shard communication, the CSLAP may be compatible with most atomic commit protocols. In the future, we can integrate this protocol with other cross-shard communication technologies.

A potential challenge in adapting the CSLAP to different network models is managing latency variations. Strategies for addressing this issue might involve introducing adaptive timeouts that adjust based on observed network conditions. This approach would help maintain system stability even in unpredictable environments.

With respect to adversary models, we will explore mechanisms for detecting and mitigating attacks from highly adaptive adversaries, and techniques such as randomized protocol steps or frequent leader rotation could improve the system’s resilience against targeted attacks. This research direction aims to increase the robustness and adaptability of the CSLAP in diverse blockchain applications.

Overall, these future research efforts will contribute to enhancing the versatility of the CSLAP and provide innovative solutions for developing resilient blockchain technologies.

## Data Availability

The code sets used and/or analysed in this study are available from the corresponding authors.

## Code availability

The code for this protocol is open source and available on github:https://github.com/zwd444456/CSLAPprotocol.
